# Sodium 4-Carboxymethoxyimino-(4-HPR) a Novel Water-Soluble Derivative of 4-Oxo-4-HPR Endowed with *In Vivo* Anticancer Activity on Solid Tumors

**DOI:** 10.3389/fphar.2017.00226

**Published:** 2017-04-26

**Authors:** Paola Tiberio, Elena Cavadini, Loredana Cleris, Sabrina Dallavalle, Loana Musso, Maria G. Daidone, Valentina Appierto

**Affiliations:** ^1^Biomarkers Unit, Department of Experimental Oncology and Molecular Medicine, Fondazione IRCCS Istituto Nazionale dei TumoriMilan, Italy; ^2^Division of Chemistry and Molecular Biology, Department of Food, Environmental and Nutritional Sciences, Università degli Studi di MilanoMilan, Italy

**Keywords:** retinoids, sodium 4-carboxymethoxyimino-(4-HPR), solid tumors, antimitotic agent, apoptosis, ROS, tubulin

## Abstract

4-oxo-*N*-(4-hydroxyphenyl)retinamide (4-oxo-4-HPR), an active polar metabolite of the synthetic retinoid *N*-(4-hydroxyphenyl)retinamide (4-HPR), was shown to exert promising antitumor activity through at least two independent mechanisms of action. Specifically, differently from 4-HPR and other retinoids, 4-oxo-4-HPR targets microtubules and inhibits tubulin polymerization causing mitotic arrest and on the other hand, analogously to the parent drug, it induces apoptosis through the activation of a signaling cascade involving the generation of reactive oxygen species (ROS). However, the potential *in vivo* use of 4-oxo-4-HPR is impaired by its poor solubility. By chemical modification of 4-oxo-4-HPR, a new class of compounds with improved solubility and *in vivo* bioavailability was obtained. We demonstrated here that, among them, the most promising molecule, sodium 4-carboxymethoxyimino-(4-HPR), was endowed with *in vitro* antitumor efficacy and entirely preserved the double mechanism of action of the parent drug in cancer cells of different histotypes. In fact, the retinoid induced the activation of the apoptotic cascade related to the generation of ROS through endoplasmic reticulum stress response and upregulation of phospho c-Jun N-terminal kinases and PLAcental Bone morphogenetic protein, leading to cell death through caspase-3 cleavage. Otherwise, sodium 4-carboxymethoxyimino-(4-HPR) caused a marked mitotic arrest coupled with multipolar spindle formation and tubulin depolymerization. To assess the compound antitumor activity, *in vivo* experiments were performed in three mouse xenograft models (ovarian and breast cancers and mesothelioma). The *in vivo* results demonstrated that retinoid administration as single agent significantly increased the survival in ovarian cancer xenografts, induced a statistically significant decrease in tumor growth in breast cancer xenografts, and caused a 30% reduction in tumor growth in a mesothelioma mouse model. Even though further studies investigating sodium 4-carboxymethoxyimino-(4-HPR) toxicity and *in vitro* and *in vivo* activities in combination with other drugs are required, the double mechanism of action of the retinoid coupled with its *in vivo* antitumor efficacy and potential low toxicity suggest a promising therapeutic potential for the compound in different solid tumors.

## Introduction

Among retinoids, natural and synthetic derivatives of vitamin A (retinol), fenretinide or *N*-(4-hydroxyphenyl)retinamide (4-HPR) (Nagy et al., 1998) has given interesting results for preneoplastic (Tradati et al., 1994; Moglia et al., 1996; Chiesa et al., 2005) and neoplastic disorders (De Palo et al., 2002; Veronesi et al., 2006). 4-oxo-*N*-(4-hydroxyphenyl)retinamide (4-oxo-4-HPR) is a polar metabolite of 4-HPR, identified by our group in plasma samples of 4-HPR-treated patients and in the culture medium of 4-HPR-treated cancer cells. 4-oxo-4-HPR elicited antiproliferative and apoptotic effects in various cancer cell lines (e.g., neuroblastoma, ovarian and breast cancer cell lines) and it was two to four times more effective than 4-HPR in inhibiting cell growth (Villani et al., 2004). Interestingly, 4-oxo-4-HPR was also effective in 4-HPR-resistant cancer cells and, in combination with 4-HPR, displayed a synergistic effect (Villani et al., 2006). Our molecular studies have demonstrated that 4-oxo-4-HPR antitumor effect was due to at least two independent mechanisms of action (Tiberio et al., 2010). Specifically, 4-oxo-4-HPR, unlike 4-HPR and other retinoids, inhibited tubulin polymerization, causing a marked accumulation of cells in mitotic phase, resulting in aberrant spindle formation (i.e., multipolar organization without loss of centrosome integrity) (Appierto et al., 2009a). On the other hand, similarly to 4-HPR, 4-oxo-4-HPR caused apoptosis through the activation of a signaling cascade starting from the generation of reactive oxygen species (ROS) and involving the endoplasmic reticulum (ER) stress response, the activation of Jun N-terminal Kinase (JNK) and the PLAcental Bone morphogenetic protein (PLAB) upregulation (Tiberio et al., 2010). The ability of 4-oxo-4-HPR to act through at least two unrelated mechanisms could provide an explanation of its higher antitumor activity compared to the parent drug thus potentially making it a promising anticancer agent able to counteract the development of drug resistance.

**FIGURE 1:**
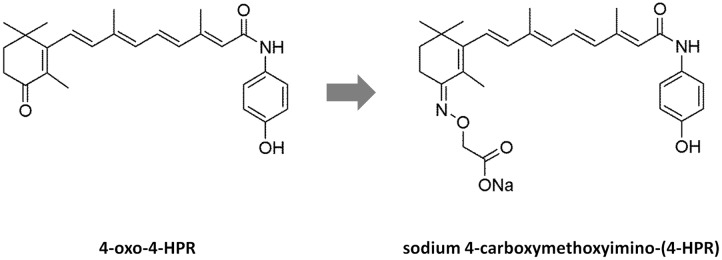
**Structures of 4-oxo-4-HPR and sodium 4-carboxymethoxyimino-(4-HPR).** The chemical structures of 4-oxo-4-HPR **(Left)** and of its new derivative **(Right)** are depicted.

However, in *in vivo* experiments conducted in nude mice (Musso et al., 2016), 4-oxo-4-HPR accumulated at the injection site and drug plasma levels reached low and variable concentrations, probably due to the poor solubility of this compound. In the attempt to improve solubility and bioavailability of 4-oxo-4-HPR, we have modified the chemical structure of the drug, obtaining a new class of compounds with higher solubility but preserving the antitumor activity of the parent drug (Musso et al., 2016). The most promising compound of this class, 3-[8-(4-Hydroxyphenylcarbamoyl)-3,7-dimethylocta-1,3,5,7-tetraenyl]-2,4,4-trimethylcyclohex-2-enylideneaminooxy sodium acetate or sodium 4-carboxymethoxyimino-(4-HPR) (**Figure 1**), has shown to induce a significant cycle arrest in G_2_-M coupled with growth inhibition and accumulation of cells in sub-G_1_ in A2780 cell line (Musso et al., 2016).

Moreover, by analyzing the *in vivo* solubility and bioavailability in a mouse model, we verified that sodium 4-carboxymethoxyimino-(4-HPR) was more soluble than 4-oxo-4-HPR, reaching 60-fold higher plasma levels, even if administered at half dose, with very low variability and without evident signs of toxicity for the mice (Musso et al., 2016).

The present study was planned to dissect the molecular mechanism of action of this new promising 4-HPR derivative and to investigate its *in vivo* antitumor activity in solid tumors in order to propose a new potential agent for cancer therapy. Here we reported that the compound exerted its antitumor activity in cancer cells of different histotypes trough the double mechanism of action of the parent drug, and displayed *in vivo* antitumor efficacy in different solid tumors.

## Materials and Methods

### Cell Lines and Reagents

Cell lines, source and culture conditions are described in **Table 1**. All cell lines were cultured in monolayer at 37°C under 5% CO_2_.

**Table 1:** Cell lines and culture conditions.

Cell line	Source	Culture medium
**Ovarian cancer:**		
A2780	Obtained from Dr. Ozols, Bethesda, MD, USA	RPMI 1640 + 10% FCS
IGROV-1	Obtained from Dr. Bénard, Villejuif, France	RPMI 1640 + 10% FCS
SKOV-3	Purchased from ATCC, Manassas, VA, USA	RPMI 1640 + 10% FCS
**Breast cancer:**		
T47-D	Obtained from Dr. R. Sutherland, Sydney, NSW, Australia	RPMI 1640 + 10% FCS + 0.25 unit/mL insulin
BT-474	Purchased from ATCC, Manassas, VA, USA	RPMI 1640 + 10% FCS
MDA-MB-231	Obtained from Dr. R. Sutherland, Sydney, NSW, Australia	RPMI 1640 + 10% FCS
**Neuroblastoma:**		
SK-N-BE	Purchased from ATCC, Manassas, VA, USA	RPMI 1640 + 10% FCS
SH-SY5Y	Purchased from ATCC, Manassas, VA, USA	RPMI 1640 + 10% FCS
SK-N-SH	Purchased from ATCC, Manassas, VA, USA	E-MEM + 10% FCS
**Mesothelioma:**		
STO	Established by our group from surgical specimens of patients who underwent surgery at INT^∗^	DMEM F12 + 10% FCS
MESO	Established by our group from surgical specimens of patients who underwent surgery at INT^∗^	DMEM F12 + 10% FCS


*FCS, fetal calf serum; ATCC, American Type Culture Collection; INT, Fondazione IRCCS Istituto Nazionale dei Tumori of Milan. ^∗^Zaffaroni et al. (2007).*

Sodium 4-carboxymethoxyimino-(4-HPR), synthesized as previously described (Musso et al., 2016), was dissolved at 10 mmol/L in DMSO prior to further dilution in culture medium and stored at -80°C in the dark. Vinblastine (Sigma–Aldrich, St. Louis, MO, USA) was dissolved at 1 mmol/L in water before further dilution in culture medium and stored at 4°C. Vitamin C (Sigma, St. Louis, MO, USA) was added to cells 1 h before Sodium 4-carboxymethoxyimino-(4-HPR) treatment.

### Growth Inhibition Assay

Cells were seeded in 96-well tissue culture plates at 7 × 10^3^ cells/well and were allowed to adhere for 24 h before treatment. Cells were grown in the presence of vehicle or sodium 4-carboxymethoxyimino-(4-HPR) at a final concentration of 0.3, 1, 3, 5, and 10 μM. Cellular growth was assessed after 72 h by sulforhodamine B (SRB) assay, as previously described (Tiberio et al., 2010). The antiproliferative activity of the compound was tested in three independent experiments with four replicate wells for each analysis.

### Determination of Reactive Oxygen Species

Intracellular production of ROS was assessed by employing the oxidation-sensitive dye 5-(and-6)-chloromethyl-2′,7′-dichlorodihydrofluorescein diacetate (CM-H2DCFDA; Molecular Probes, Inc., Eugene, OR, USA) as described previously (Villani et al., 2006) Briefly, 8 × 10^5^ cells/well were plated in six-well cell culture plates and incubated for 4 h in the presence of 3 and 5 μM sodium 4-carboxymethoxyimino-(4-HPR) and/or 100 μM vitamin C. Medium was discarded and, under low light conditions, replaced with 50 μM CM-H2DCFDA in whole medium for 20 min at 37°C. Cells were harvested, transferred to foil-wrapped tubes and analyzed immediately by flow cytometry. The ROS generation was tested in three independent experiments with three replicates for each sample in each analysis.

### Immunoblot Analysis

Proteins were extracted by using sodium dodecyl sulfate (SDS) sample buffer (62.5 mM Tris–HCl [pH 6.8], 2% SDS) containing 1 mM phenylmethylsulfonyl fluoride, 10 μg/mL pepstatin, 12.5 μg/mL leupeptin, 2 μg/mL aprotinin, 1 mM sodium orthovanadate, and 1 mM sodium molybdate. Proteins were processed for western immunoblotting as previously described (Appierto et al., 2004). The following antibodies used for immunoblotting were purchased from the indicated suppliers and used according the manufacturer testing conditions: PLAB and pJNK from Santa Cruz Biotechnology (Santa Cruz, CA, USA); cleaved caspase-3 and pEIF2α from Cell Signaling (Beverly, MA, USA); and GAPDH, α-tubulin, and actin from Sigma–Aldrich. Quantification of pJNK and pEIF2α band intensities was performed using the ImageJ software freely available at http://rsb.info.nih.gov/ij/. Values were normalized by actin band intensity.

### Tubulin Polymerization Assay

Cells were seeded in 24 mm Petri dishes and the day after were exposed to different concentration of sodium 4-carboxymethoxyimino-(4-HPR) and vinblastine and 24 h later processed for the tubulin polymerization assay as previously described (Cappelletti et al., 2004; Appierto et al., 2009a). Cytosolic and cytoskeletal-associated proteins were separated by SDS–PAGE and tubulin distribution was analyzed by immunoblotting using α-tubulin antibody (Sigma–Aldrich).

### Cell Cycle Analysis

Analyses of cell cycle distribution were conducted as previously described by our group (Tiberio et al., 2010) after 24 h of treatment with 3 and 5 μM sodium 4-carboxymethoxyimino-(4-HPR) and/or 100 μM vitamin C. Cell cycle analysis was performed using FACScan flow cytometer (Becton Dickinson, San Jose, CA, USA). The percentage of cells in different phases of cell cycle was determined by ModFit LT cell cycle analysis software (Verity Software House, Topsham, ME, USA), considering only cells with DNA content ≥2n. Apoptotic cells were identified as a sub-G1 population (DNA content <2n).

### Immunofluorescence Analysis

Cells, grown on glass coverslip slides in 24 mm Petri dishes, were fixed in 100% methanol at -20°C for 7 min, washed with PBS and then blocked at room temperature for 1 h in 3% BSA/ 0.1% (v/v) Triton X-100/PBS. Cells were incubated overnight at 4°C with the primary antibody mouse anti-α-tubulin (Sigma–Aldrich), washed three times with PBS, and then incubated for another hour at room temperature with the secondary antibody anti-mouse Alexa 488 (Molecular Probes, Inc., Eugene, OR, USA), washed thrice with PBS and stained with Hoechst 33342. Slides were mounted with Mowiol (Calbiochem, San Diego, CA, USA), and viewed with a fluorescence microscope [images were recorded with a Spot Insight digital camera (Delta Sistemi, Rome, Italy) equipped with a system of image analysis (IAS 2000; Delta Sistemi)].

### Mouse Xenograft Models and *In Vivo* Treatments

All mice were purchased from Charles River (Calco, Italy) and maintained in laminar-flow rooms or individually ventilated cages at constant temperature and humidity, with food and water administered *ad libitum*. All the *in vivo* experiments were approved by the Ethics Committee for Animal Experimentation of the Fondazione IRCCS Istituto Nazionale Tumori of Milan according to National and Institutional guidelines.

Three different mouse models have been employed: peritoneal mesothelioma (STO cells), ovarian carcinoma (IGROV-1 *in vivo* cell line, growing mainly as ascites and maintained by i.p. serial passages of ascitic cells) and breast (MDA-MB-231 cells) cancer. As regards to mesothelioma, nude mice were inoculated s.c. with 10 × 10^6^ STO cells, and treatment with sodium 4-carboxymethoxyimino-(4-HPR) began 1 day after tumor cell inoculation (doses: 30 and 60 mg/kg i.p.; 5 days/week for 5 weeks; end of treatment: day 32). Tumor growth in the control and treated groups were evaluated, and the differences were statistically analyzed.

For the ovarian cancer model, nude mice were inoculated i.p. with 2.5 × 10^6^ IGROV-1 cells, and treatment began 1 day after tumor cell inoculation (doses: 30, 60, and 90 mg/kg i.p.; 5 days/week for 3 weeks; end of treatment: day 18). Mice with ovarian cancer were treated for only 3 weeks because IGROV-1 tumor is characterized by rapid growth and accumulation of ascites. For ethical reasons, mice were sacrificed as ascites became evident (before impending death) and 50% of control mice were sacrificed within day 20. The day of sacrifice was considered as the day of death for calculating and statistically analyzing the survival time of each group. In order to also evaluate ascitic growth (viable tumor cells were counted using the trypan blue exclusion assay) and tumor weight (TW), six additional mice were used: three treated with 60 mg/kg i.p. of sodium 4-carboxymethoxyimino-(4-HPR) (we decided to use an intermediate dosage) and three control mice. Ascitic fluid and tumor tissues were collected during the last week of treatment.

As third cancer model, 5 × 10^6^ human breast cancer cells (MDA-MB-231) were resuspended in PBS and mixed 1:1 (v/v) with Matrigel (BD Biosciences, Bedford, MA, USA) and were inoculated into the right second mammary fat pad (m.f.p.) of NOD/SCID mice under anesthesia (ketamine–xylazine). Treatment with the drug began 1 day after tumor cell inoculation (dose: 90 mg/kg; 4 days/week for 7 weeks; end of treatment: day 45). We decided to adopt for the breast cancer model a different schedule of treatment (i.e., 4 instead of 5 days/week), taking in consideration the different mouse strain employed and a long-term treatment hypothesized for this experiment. Tumor growth in the control and treated groups were evaluated, and the differences were statistically analyzed.

For all the *in vivo* experiments, the drug was first dissolved in DMSO, diluted in sterile water at final DMSO concentration of 5% (higher dose), and then diluted in sterile water to obtain the other doses. It was administered by i.p. route and delivered at 10 mg/kg of body weight. Control mice were treated with the same solvent as used to dissolve the sodium 4-carboxymethoxyimino-(4-HPR). The animals were examined thrice a week to check tumor growth, body weight and any signs of toxicity. TW was calculated by the formula TW (g) = (*d*^2^ × *D*) / 2 where *d* and *D* are the shortest and the longest diameters, respectively, measured in centimeters. All the experiments were conducted in duplicate, obtaining comparable results.

### Statistical Analysis

Differences between mean values were assessed by two-tailed Student’s *t*-test and for survival analysis data were compared with the log-rank test. The analyses were performed using GaphPad Prism v.5.02 (GraphPad Software, La Jolla, CA, USA). *P*-values < 0.05 were considered as statistically significant.

## Results

### Sodium 4-Carboxymethoxyimino-(4-HPR) Inhibits Cell Growth of Human Cancer Cell Lines of Different Histotypes

The improvement in solubility obtained through the chemical modification of 4-oxo-4-HPR (Musso et al., 2016), prompted us to investigate the antitumor activity of this new derivative. The growth-inhibitory effect of sodium 4-carboxymethoxyimino-(4-HPR) was tested in a panel of human cancer cell lines of different histotypes. The 50% effective cytotoxic concentrations (EC_50_) after 72 h of treatment were calculated and reported in **Table 2**. The compound showed an ability to inhibit the cell growth similar to that of the parent drug 4-oxo-4-HPR (Villani et al., 2006), in ovarian and breast cancer cell lines and in peritoneal mesothelioma and neuroblastoma cells.

**Table 2:** Antiproliferative activity of sodium 4-carboxymethoxyimino-(4-HPR) on solid tumor cell lines of different histotypes.

Tumor cell lines	EC_50_ (μM)
**Ovary**	
A2780	2.78
IGROV-1	3.82
SKOV-3	5.96
**Breast**	
T47-D	3.95
MDA-MB-231	3.97
BT-474	3.69
**Neuroblastoma**	
SK-N-BE	1.55
SK-N-SH	1.9
SH-SY5Y	1.3
**Mesothelioma**	
STO	1.48
MESO	1.28


Overall, these results indicate that sodium 4-carboxymethoxyimino-(4-HPR) is a retinoid derivative effective in inhibiting proliferation of cancer cells of different histotypes.

### Sodium 4-Carboxymethoxyimino-(4-HPR) Maintains the Mechanism of Action of 4-oxo-4-HPR in A2780 Cells

**FIGURE 2:**
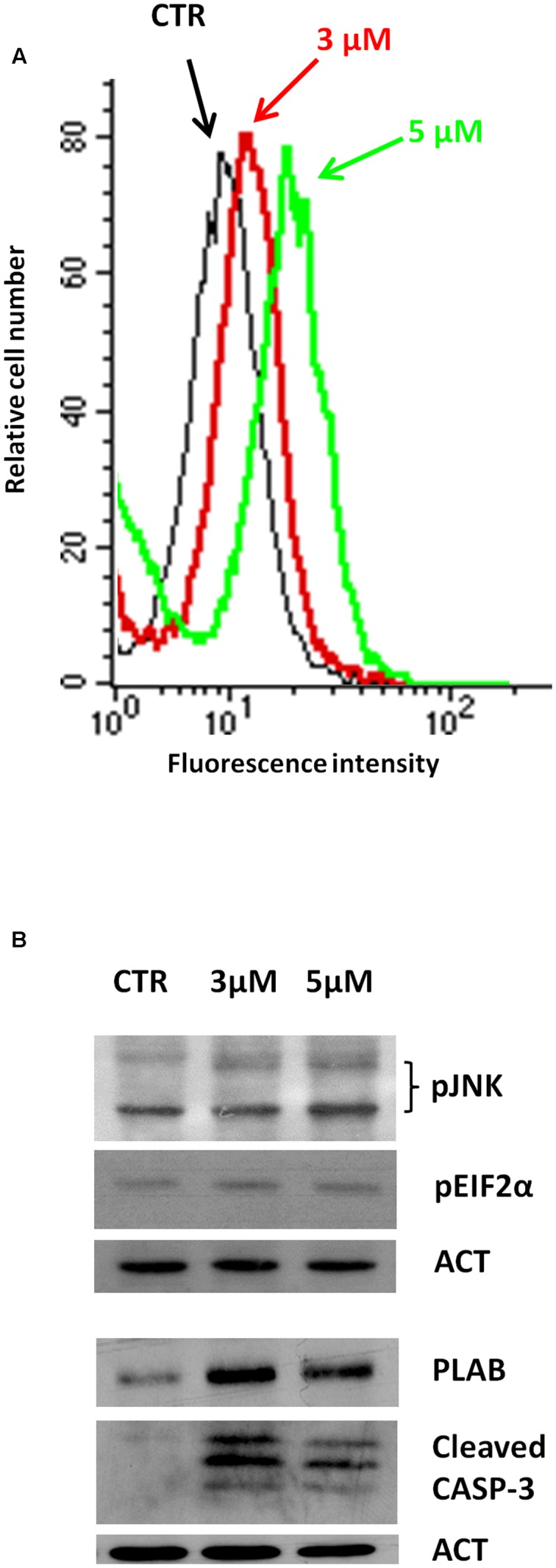
**Sodium 4-carboxymethoxyimino-(4-HPR) is able to activate the ROS-related apoptotic cascade.** **(A)** Analysis of ROS production in A2780 cells treated for 4 h with 3 and 5 μM of sodium 4-carboxymethoxyimino-(4-HPR). The graph shows representative flow cytometry fluorescence profiles in different conditions of treatment (one representative experiment of three). **(B)** A2780 cells treated for 24 h with 3 and 5 μM of sodium 4-carboxymethoxyimino-(4-HPR) were subjected to western blot analysis for the expression of pJNK, pEIF2α, PLAB and cleaved caspase-3. As a control for loading, the blots were incubated with actin antibody.

**FIGURE 3:**
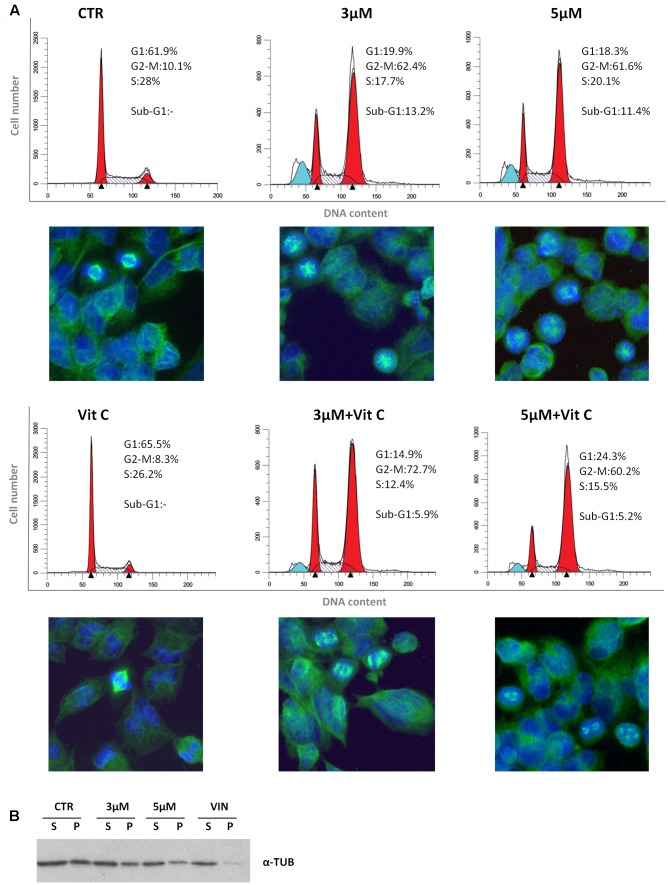
**Sodium 4-carboxymethoxyimino-(4-HPR) is endowed with antimitotic activity.** **(A)** Flow cytometric analysis of propidium iodide-stained A2780 cells treated for 24 h with vehicle or 3 and 5 μM of sodium 4-carboxymethoxyimino-(4-HPR) with or without 100 μM vitamin C. Numbers in the figure indicate the percentage of cells in the phase of cell cycle. One experiment representative of three is shown. On the bottom of each histogram, immunofluorescence analysis with α-tubulin antibody (green) of cells treated in the same way. Nuclear morphology was visualized by staining with Hoechst 33342 (blue). Photos were taken at 40× magnification. **(B)** Western blot analysis of soluble cytosolic (*s*) or polymerized (*p*) tubulin in A2780 cells after 24 h of exposure to vehicle, sodium 4 carboxymethoxyimino-(4-HPR), and vinblastine (tubulin depolymerizing agent).

We have recently reported that the chemical modification performed on 4-oxo-4-HPR and leading to sodium 4-carboxymethoxyimino-(4-HPR) did not impair the antimitotic activity of the parental drug, in terms of cell cycle arrest and formation of multipolar spindles (Musso et al., 2016). In the present study we deeper dissected the activity of the compound to investigate whether this drug entirely preserved the double mechanism of action previously described for 4-oxo-4-HPR (Appierto et al., 2009a; Tiberio et al., 2010). To this aim, we first analyzed whether sodium 4-carboxymethoxyimino-(4-HPR) induced the generation of ROS in A2780, a human ovarian carcinoma cell line previously used as *in vitro* model to investigate the mechanism of action of the parental drug (Appierto et al., 2009a; Tiberio et al., 2010). The analysis revealed that the molecule caused the generation of ROS in a dose-dependent manner (**Figure 2A**). Treatment with both 3 and 5 μM 4-carboxymethoxyimino-(4-HPR) for 24 h caused a twofold increase in JNK phosphorylation, a slight induction of pEIF2α (1.5-fold over control for both drug doses), marked PLAB upregulation and caspase-3 cleavage (**Figure 2B**), suggesting that sodium 4-carboxymethoxyimino-(4-HPR) was able to induce the activation of the ROS-related apoptotic cascade described for its parental drug (Tiberio et al., 2010). On the other hand, the treatment led to a marked dose-dependent accumulation of cells in the G_2_-M phases, associated with abnormal mitotic spindles with loss of normal bipolarity and formation of multipolar spindles, as revealed by immunofluorescence labeling for α-tubulin (**Figure 3A**).

To investigate whether the sodium 4-carboxymethoxyimino-(4-HPR)-induced mitotic arrest and formation of multipolar spindles were related to the ROS generation, the effects of the antioxidant vitamin C on cell cycle perturbation and formation of aberrant spindles induced by the compound were analyzed. In A2780 cells exposed for 24 h to 5 μM 4-carboxymethoxyimino-(4-HPR), the addition of 100 μM vitamin C abrogated ROS generation (**Supplementary Figure S1**) and caused a reduction of sub-G1 population but did not prevent the accumulation of G_2_-M cell population or the formation of aberrant spindles (**Figure 3A**). The results indicated that in A2780 cells, the mitotic arrest induced by 4-carboxymethoxyimino-(4-HPR) was independent from ROS generation.

**FIGURE 4:**
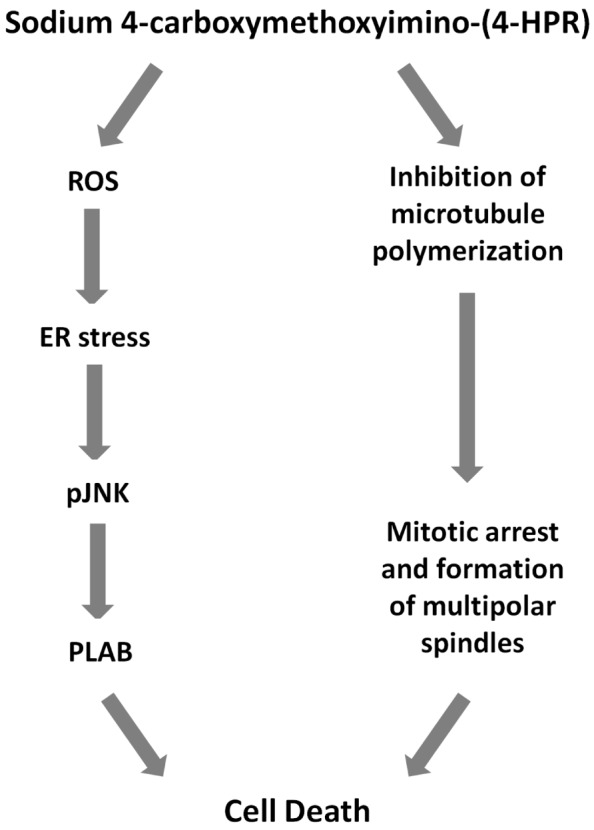
**Mechanisms of action of sodium 4-carboxymethoxyimino-(4-HPR).** Scheme showing proposed cascade of events involved sodium 4-carboxymethoxyimino-(4-HPR)-induced growth inhibitory effect.

Finally, we investigated the effects of the 4-oxo-4-HPR derivative on the microtubule system of A2780 cells treated for 24 h with two different concentrations of the drug (i.e., 3 and 5 μM). Similarly to 4-oxo-4-HPR (Appierto et al., 2009a), sodium 4-carboxymethoxyimino-(4-HPR) decreased the polymerized portion of tubulin in a dose-dependent manner, as revealed by western blot analysis of free and polymerized tubulin (**Figure 3B**). Thus, the aberrant formation of mitotic spindles caused by the drug could be ascribed to the inhibition of microtubule polymerization induced by the retinoid in cultured cells, as previously described for 4-oxo-4-HPR (Appierto et al., 2009a). Taken together, our data indicated that sodium 4-carboxymethoxyimino-(4-HPR) entirely maintained the double mechanism of action of its parental drug in A2780 cells (**Figure 4**).

### Sodium 4-Carboxymethoxyimino-(4-HPR) Acts through a Double Mechanism of Action in Different Cancer Cell Lines

**FIGURE 5:**
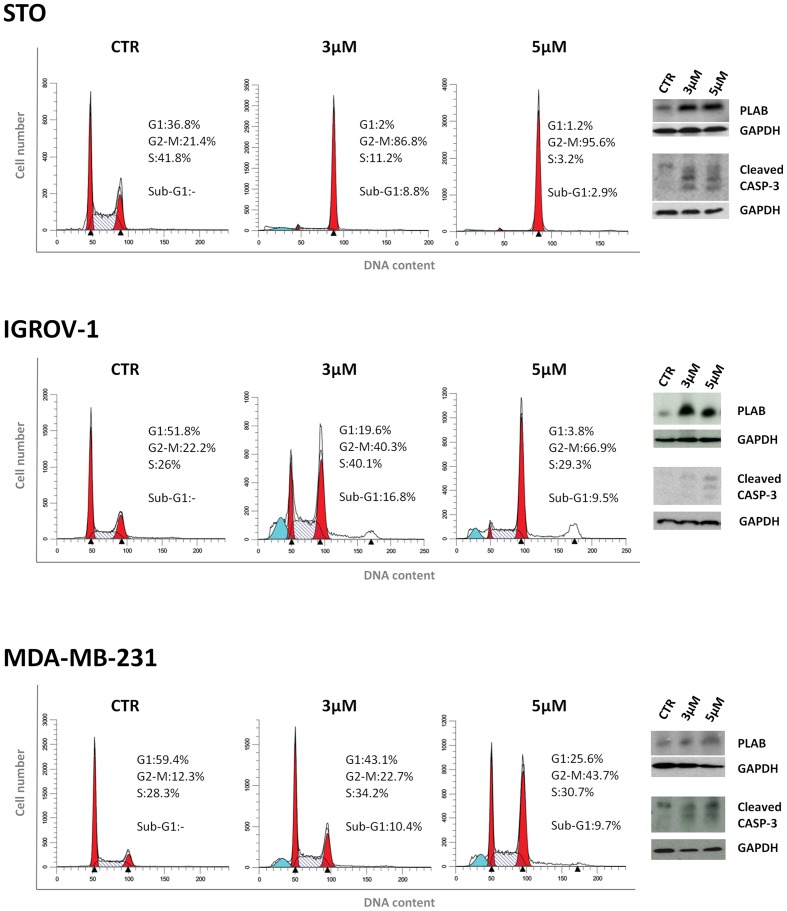
**Sodium 4-carboxymethoxyimino-(4-HPR) acts through a double mechanism of action in different cancer cell lines.** **Left**, flow cytometric analysis of propidium iodide-stained tumor cells treated with vehicle or 3 and 5 μM of sodium 4-carboxymethoxyimino-(4-HPR) for 24 h. Numbers in the figure indicate the percentage of cells in the phase of cell cycle. One experiment representative of three is shown. **Right**, the same treated cells were subjected to western blot analysis for the expression of PLAB and cleaved caspase-3. As a control for loading, the blots were incubated with GAPDH antibody.

We next investigated whether the two mechanisms of action of 4-carboxymethoxyimino-(4-HPR) described in A2780 were recapitulated also in other human cancer cells responsive to the retinoid. Specifically, we analyzed the upregulation of PLAB and the cleavage of caspase-3 for the activation of the ROS-induced apoptotic cascade and the accumulation in G_2_-M phases of the cell cycle for the antimitotic activity in STO (mesothelioma cells derived from a patient’s surgical specimen), IGROV-1 (ovarian cancer cell line), and MDA-MB-231 (triple negative breast cancer cell line) cells. In the three analyzed cell lines, the treatment with the drug induced both PLAB upregulation and caspase-3 cleavage and a marked G_2_-M cell cycle arrest (**Figure 5**). The results confirmed that the distinctive feature of sodium 4-carboxymethoxyimino-(4-HPR) to have a double mechanism of action in solid tumors was not restricted to A2780 cells but represented a distinctive mode of action of the drug (**Figure 4**).

### Sodium 4-Carboxymethoxyimino-(4-HPR) Is Endowed with *In Vivo* Antitumor Activity on Different Solid Tumor Models

**FIGURE 6:**
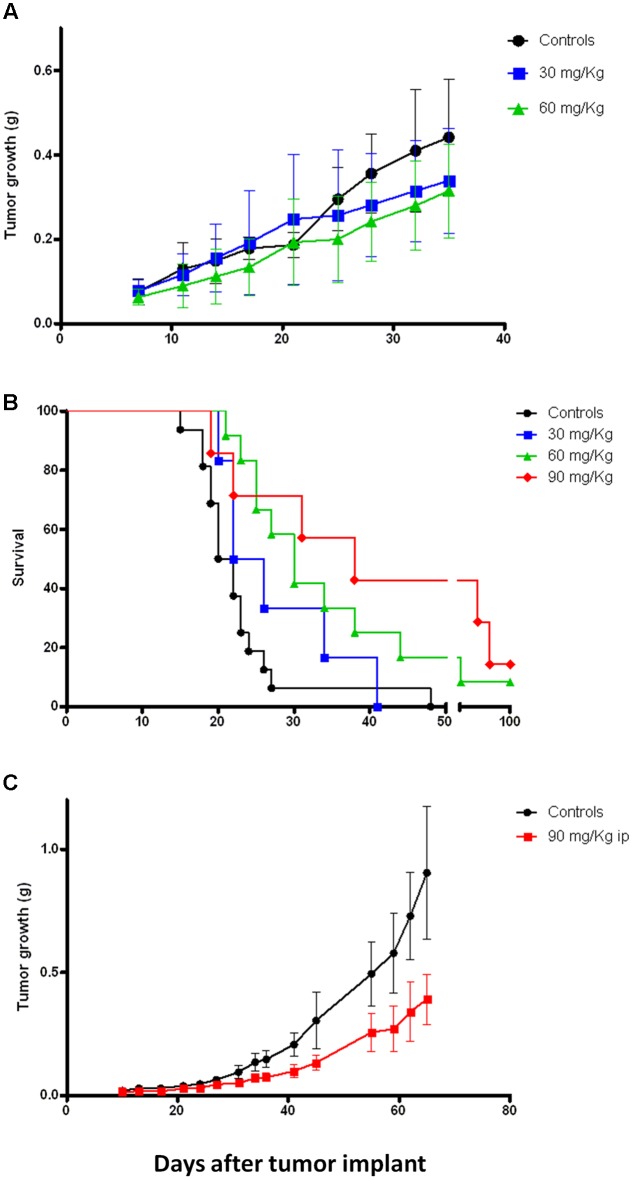
**Sodium 4-carboxymethoxyimino-(4-HPR) exerts *in vivo* antitumor activity on different tumor models.** **(A)** Tumor growth curves of mice xenografted with human mesothelioma cells (STO) and treated 5 days/week for 5 weeks (end of treatment: day 32) with vehicle or 30 and 60 mg/kg of sodium 4-carboxymethoxyimino-(4-HPR). **(B)** Survival analysis of mice xenografted with human ovarian cancer cells (IGROV-1) and treated 5 days/week for 3 weeks (end of treatment: day 18) with vehicle or 30, 60, and 90 mg/kg of sodium 4-carboxymethoxyimino-(4-HPR). **(C)** Tumor growth curves of mice xenografted with human triple negative breast cancer cells (MDA-MB-231) and treated for 4 days/week for 7 weeks (end of treatment: day 45) with vehicle or 90 mg/kg of sodium 4-carboxymethoxyimino-(4-HPR).

Previous *in vivo* preliminary study demonstrated that, compared to 4-oxo-4-HPR, sodium 4-carboxymethoxyimino-(4-HPR) reached 60-fold higher plasma levels, with extremely low variability and without apparent signs of toxicity for the mice (Musso et al., 2016). This promising evidence led us to investigate the antitumor activity of the compound in mouse models. Specifically, mice xenografted with human mesothelioma (STO), ovarian cancer (IGROV-1) and breast cancer (MDA-MB-231) cells were employed. Mice with mesothelioma xenografts were treated 5 days/week for 5 weeks with 30 or 60 mg/kg i.p. of sodium 4-carboxymethoxyimino-(4-HPR). As shown in **Figure 6A**, the treatment with the retinoid reduced the tumor growth at both doses, although a statistical significance was not reached. As regards to ovarian model, the mice were treated 5 days/week for 3 weeks with 30, 60, and 90 mg/kg i.p. of retinoid. Survival, TW and the number of ascitic cells were evaluated. At the doses of 60 and 90 mg/kg, the drug significantly increased the survival of the mice inoculated with IGROV-1 cells (*p* < 0.01; **Figure 6B**). Consistently, solid tumor mass and the number of ascitic cells were reduced in treated mice compared to control group of 62% (on average g: 0.59 ± 0.13 SEM and 0.22 ± 0.055 SEM, respectively) and 90% (on average cells: 165.5 × 10^6^ ± 68.40 × 10^6^ SEM and 20.13 × 10^6^ ± 15.74 × 10^6^ SEM, respectively).

Based on the results obtained in the previous models, we decided to test a single dose (90 mg/kg) for *in vivo* experiments on the breast cancer model. Specifically, the mice were subjected for 7 weeks to a 4 days/week treatment with the compound. As shown in **Figure 6C**, the retinoid significantly reduced the tumor growth of the triple negative breast cancer model (*p* = 0.01).

In all the experiments the compound administration was well tolerated with maximum body weight loss of 9% restricted to the first 3 days of treatment.

Overall our *in vivo* data demonstrated that sodium 4-carboxymethoxyimino-(4-HPR) deserves further investigations as a new potential anticancer drug able to exert its activity on different solid tumor types.

## Discussion

4-oxo-4-HPR, a polar 4-HPR metabolite, was shown to be a potential promising antitumor agent (Appierto et al., 2009a; Tiberio et al., 2010) even though its poor solubility limited *in vivo* testing (Musso et al., 2016). By chemical modification of 4-oxo-4HPR we obtained a new compound, sodium 4-carboxymethoxyimino-(4-HPR), with improved solubility and *in vivo* bioavailability (Musso et al., 2016).

The compound was effective in inhibiting the growth of a panel of cancer cells of different histotypes, such as ovarian and breast cancers, neuroblastoma and mesothelioma. The antiproliferative activity was comparable to that observed for 4-oxo-4-HPR and prompted us to evaluate whether sodium 4-carboxymethoxyimino-(4-HPR) entirely preserved the mechanism of action of the parent drug. We previously demonstrated that the 4-HPR derivative induced apoptosis via the signaling cascade activated also by 4-HPR (ROS → ER stress → JNK → PLAB) but, differently from the latter, also acted as an antimicrotubule agent (Appierto et al., 2009a,b; Tiberio et al., 2010). By investigating mechanisms of action of the new compound in A2780 cell line we found that the treatment with the retinoid caused the generation of ROS in a dose-dependent manner. As a downstream effect, sodium 4-carboxymethoxyimino-(4-HPR) induced the activation of the entire ROS-related apoptotic cascade through the ER stress response (determined by the phosphorylation of EIF2α) and the upregulation of pJNK and PLAB, leading to caspase-3 cleavage. The activation of this apoptotic pathway was not restricted to A2780 cell line but was observed also in STO, IGROV-1 and MDA-MB-231 cells. Similarly, in all tested cancer cell lines, the compound induced a marked accumulation of cells in G_2_-M phase in a dose-dependent manner. As previously demonstrated for 4-oxo-4-HPR, the cell cycle arrest was coupled with multipolar spindle formation and tubulin depolymerization. Noteworthy, the mitotic arrest and the coupled formation of multipolar spindles induced by sodium 4-carboxymethoxyimino-(4-HPR) were independent from the ROS-related signaling cascade. In fact, inhibition of the ROS generation by vitamin C did not prevent the ability of the compound to exert its antimicrotubule activity.

Overall, our *in vitro* data demonstrated that sodium 4-carboxymethoxyimino-(4-HPR) entirely preserve the double mechanism of action described for its parent drug (**Figure 4**). The antimitotic activity of the drug makes it potentially promising for the treatment of high-proliferating solid tumors (such as, triple negative breast cancer). Moreover, we could speculate that its ability to act through, at least, two unrelated pathways could overcome the occurrence of resistance mechanism. However, further preclinical studies are definitely needed to investigate whether 4-carboxymethoxyimino-(4-HPR) could be effective in the treatment of drug-resistant tumors and, in order to design effective drug combination, to investigate how the two pathways activated by the compound interact.

The antimicrotubule activity is a very atypical mechanism of action for a retinoid. In fact, to the best of our knowledge, among retinol derivatives only 4-oxo-4-HPR and its new derivative are able to directly target tubulin. We previously hypothesized that the introduction of a keto group in position 4 of the cyclohexene ring of 4-HPR, leading to 4-oxo-4-HPR, was responsible for its antimitotic activity. However, sodium 4-carboxymethoxyimino-(4-HPR) has a different chemical group at the same position (**Figure 1**). Thus, we could speculate here that the presence of polar groups at position 4 has a key role for antitubulin activity.

Taking advantage of the previously demonstrated improved bioavailability of the new derivative compared to 4-oxo-4-HPR (Musso et al., 2016) we performed *in vivo* experiments in three different mouse models (mesothelioma, ovarian, and breast cancers) to assess its antitumor effect. The retinoid induced statistically significant increase in survival and decrease in tumor growth in ovarian (IGROV-1) and triple negative breast cancer (MDA-MB-231) models, respectively. Interestingly, the treatment efficacy obtained on the ovarian and breast cancer models persisted several days after treatment cessation, allowing us to speculate that an intermittent treatment schedule might be effective for this compound to counteract tumor growth.

The treatment with sodium 4-carboxymethoxyimino-(4-HPR) caused a 30% reduction in tumor growth in a mesothelioma mouse model but this effect was not statistically significant. In spite of its *in vitro* doubling time, very similar to the other tumor models (STO 24 h; IGROV-1 26 h; MDA-MB-231 22 h), STO xenografts, contrary to the IGROV-1 and MDA-MD-231 xenografts displayed high variability in tumor growth, thus plausibly affecting the evaluation of the statistical significance of the test.

Considering that in clinical trials it has been found that retinol derivatives (such as 4-HPR) cause less toxicity than the classic chemotherapy agents (Miller, 1998), we could hypothesize that a similar lack of severe side effects could be obtained with sodium 4-carboxymethoxyimino-(4-HPR) as well. In addition, the derivative was obtained through a modification of the chemical structure of 4-oxo-4-HPR which was initially discovered in plasma samples of women enrolled in a phase III clinical trial and treated with 4-HPR for 5 years, that experienced only nyctalopia (decreased night vision) as side effect (Veronesi et al., 2006; Zanardi et al., 2006; Cazzaniga et al., 2012). Although tolerability experiments are definitely needed, based on the above-mentioned information and on our preliminary *in vivo* observations, we expect that the toxicity of sodium 4-carboxymethoxyimino-(4-HPR) could be mild.

Notably, the demonstrated efficacy of the present drug against ovarian and triple negative breast cancers could have important clinical implications since these types of tumors are fast growing, with very poor prognosis and usually treated with classical chemotherapy drugs inducing severe side effects (Partridge et al., 2001; Edwards, 2003; Markman, 2003; McWhinney et al., 2009; De Iuliis et al., 2015), thus there is an urgent need to identify novel anticancer agents and new combination therapies effective and non-toxic. In this context, also considering the fast growth of triple negative breast cancer, we could hypothesize to test sodium 4-carboxymethoxyimino-(4-HPR) in combination with other antimitotic agents currently used to treat this type of tumor (such as taxanes) in order to definitely impair the cell cycle division thus leading to cell death.

## Conclusion

The present study demonstrated, for the first time, that sodium 4-carboxymethoxyimino-(4-HPR) is endowed with both *in vitro* and *in vivo* antitumor activity in different solid tumors. Its double mechanism of action coupled with its *in vivo* antitumor activity and potential low toxicity suggest its possible use as anticancer drug for different tumors and, specifically, for actively proliferating cancers. Further studies investigating its toxicity and *in vitro* and *in vivo* activities in combination with other drugs are definitely required to understand whether the new 4-oxo-4HPR derivative has an actual therapeutic potential.

## Author Contributions

MGD and VA conceived and designed the experiments. PT, EC, LC, and LM performed the experiments. PT, SD, MGD, and VA wrote the paper. All authors analyzed the data, critically revised and approved the manuscript.

## Conflict of Interest Statement

The authors declare that the research was conducted in the absence of any commercial or financial relationships that could be construed as a potential conflict of interest. Patent international application no. PCT/EP2015/071178.

## Supplementary Material

**FIGURE S1 | Vitamin C abrogates 4-carboxymethoxyimino-(4-HPR)-ROS generation.** Analysis of ROS production in A2780 cells treated for 4 h with 3 and 5 μM sodium 4-carboxymethoxyimino-(4-HPR), with or without 100 μM vitamin C. The graphs show representative flow cytometry fluorescence profiles in different conditions of treatment (one representative experiment of three).
