# Supplementary figures and images for: Sodium 4-Carboxymethoxyimino-(4-HPR) a Novel Water-Soluble Derivative of 4-Oxo-4-HPR Endowed with In Vivo Anticancer Activity on Solid Tumors
Source: Front Pharmacol. 2017 Apr 26;8:226. doi: 10.3389/fphar.2017.00226 (PMC5405676; doi:10.3389/fphar.2017.00226)

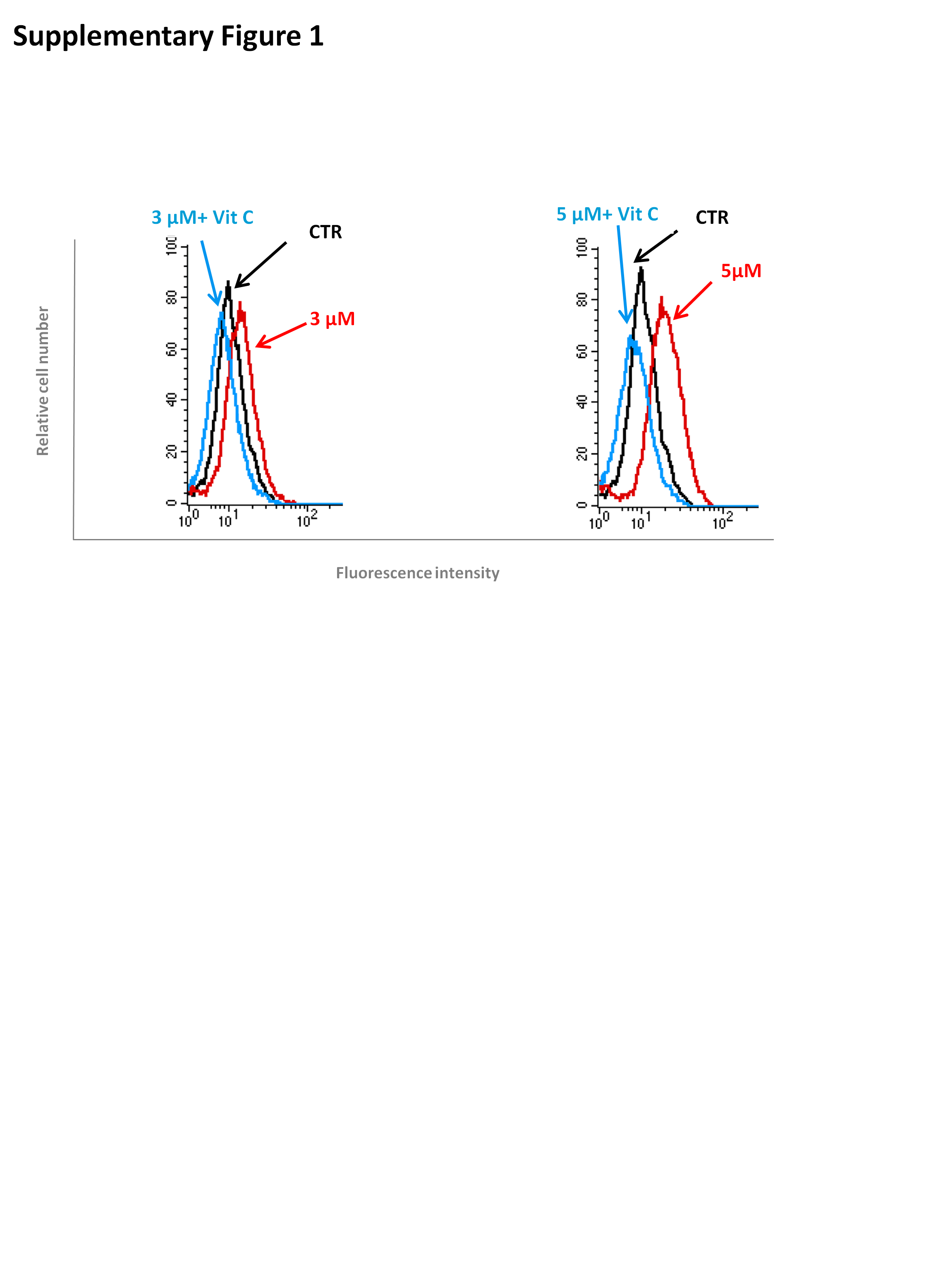

Supplement: Supplementary file 1 [file Image_1.TIF]
